# In Vitro and In Silico Analysis of the Bindings between Legacy and Novel Per- and Polyfluoroalkyl Substances and Human Serum Albumin

**DOI:** 10.3390/toxics12010046

**Published:** 2024-01-08

**Authors:** Yuqing Wu, Jia Bao, Yang Liu, Xin Wang, Xinyi Lu, Ke Wang

**Affiliations:** 1 School of Environmental and Chemical Engineering, Shenyang University of Technology, Shenyang 110870, China; 2 Department of Clinical Nutrition, Second Affiliated Hospital of Dalian Medical University, Dalian 116023, China

**Keywords:** PFASs, alternatives, human serum albumin, binding, fluorescence spectroscopy, molecular docking

## Abstract

Per- and polyfluoroalkyl substances (PFASs) are emerging contaminants of concern that can enter the human body through a variety of pathways and thereby cause harmful effects. Exposure of pregnant women to PFASs could even affect both the mother and the child. Human serum albumin (HSA) is considered to be the primary transport protein for a variety of substances in body fluids. It can be bound to different contaminants and might result in possible effects on human health. Yet, few studies are available on the binding affinity of legacy PFASs and their novel alternatives to HSA. In this study, the binding mechanisms of HSA to both legacy PFASs and their novel alternatives were investigated using fluorescence spectroscopy, together with further molecular docking. The results show that all the target PFASs were statically quenched against HSA with binding ratios of 1:1. The binding constants of long-chain PFASs and novel alternatives of perfluoroalkanesulfonic acids (PFSAs) were greater than 10^2^, whereas those of short-chain PFASs alternatives and novel alternatives of perfluorocarboxylic acids (PFCAs) were less than 10^2^. In general, the binding affinities of PFCAs on HSA were less than that of PFSAs, while the binding affinities of short-chain PFASs alternatives on HSA were smaller than those of long-chain PFASs and their novel alternatives. Therefore, bindings to HSA could be considered as an important influencing factor for the bioaccumulation of legacy and novel PFASs in the human body.

## 1. Introduction

Per- and polyfluoroalkyl substances (PFASs), which are a family of chemical substances containing one or more perfluoroalkyl moieties (–C_n_F_2n+1_–), have been used globally as highly effective surfactants and surface protectants for the past seventy years [1]. Particularly, strong perfluoroalkyl moieties have unique properties, including exceptional resistance to environmental and biodegradation, thermal and chemical stability to oxidation, photolysis, and hydrolysis reactions, as well as hydrophobic and oleophobic properties [2]. Long-chain perfluoroalkyl carboxylic acids (PFCAs) (containing seven or more perfluorinated carbons) and perfluoroalkanesulfonic acids (PFSAs) (containing six or more perfluorinated carbons) are ubiquitous around the globe and thus have received much attention [3,4]. Two decades ago, perfluorooctane sulfonate (PFOS) was found for the first time in the blood of wild animals and even in human blood [5,6]. Studies have shown that long-chain PFASs are potentially toxic and may cause reproductive and developmental defects, hepatotoxicity, neurotoxicity, and immunotoxicity [7,8]. In 2009, PFOS, its salts, and its precursor perfluorooctane sulfonyl fluoride (PFOSF) were restricted globally [9]. Furthermore, another two long-chain PFASs, perfluorooctanoic acid (PFOA) and perfluorohexane sulfonic acid (PFHxS), their salts, and related compounds were phased out from international productions in 2019 and 2022, respectively [10,11].

Since the ban on the production and application of long-chain PFASs, a number of alternatives have been commercially developed. These alternatives have similar fluorinated chain structures, such as short-chain PFASs and polyfluorinated ethers [12,13]. Recently, changes have been observed in the human body, with these alternatives reaching cumulative levels in some cases. This suggests that humans have been exposed to these emerging PFASs. These alternatives have higher environmental stability and mobility compared to legacy PFASs [14,15], and they could further migrate into the environment, become widespread, and accumulate in the environment and organisms [16,17,18,19]. For instance, perfluoropropane sulfonate (PFPrS), perfluorobutane sulfonate (PFBS), and perfluorobutanoic acid (PFBA) have emerged as short-chain alternatives for long-chain PFASs. In recent years, 1,1,2,2,3,3,3–heptafluoropropoxy propanoic acid (HFPO–DA; trade name ‘Gen–X’) and sodium dodecafluoro-3H–4,8–dioxanonanoate (NaDONA) have been produced as novel alternatives for long-chain PFOA and were subsequently detected in surface waters worldwide [20,21,22,23]. Similarly, chlorinated polyfluorinated ether sulfonates (Cl–PFESAs) are also emerging issues of concern, e.g., 9–chlorohexadecafluoro–3–oxanone–1–sulfonic acid (9Cl–PF3ONS; also known as 6:2 Cl–PFESA or the trade name trade name ‘F–53B’), and 11–chloroeicosafluoro–3–oxaundecane–1–sulfonic acid (11Cl–PF3OUdS; also known as 8:2 Cl–PFESA), and have been used as commercial mist suppressants in the electroplating industry as novel alternatives for long-chain PFOS [23], which have also been widely present in Chinese surface waters recently [24].

Human serum albumin (HSA) is a globular protein that is a single peptide chain consisting of 585 amino acid residues. HSA has 30 phenylalanine residues, 35 cysteine residues, 18 tyrosine residues, and one tryptophan residue. The N-terminal is an aspartic acid residue and the C-terminal is a leucine residue. A sulfhydryl group is present at position 34 of the single peptide chain. These amino acid residues play a very important role in maintaining the spatial structure of HSA [25]. Smeltz et al. [26] evaluated the in vitro human plasma protein binding (PPB) of 71 PFASs through ultracentrifugation and liquid chromatography–mass spectrometry analysis. The results revealed that perfluoroalkanoyl chlorides and PFCAs with 6–10 carbons were the highest bound PFASs, with similar median values for alkyl, ether, and polyethylene PFCAs. Pan et al. [27] demonstrated that HSA binding to pollutants could affect the efficiency of placental transfer. Experiments have shown that umbilical cord serum albumin can promote placental transfer of PFASs, whereas maternal serum albumin can reduce the efficiency of transfer. Previous studies have shown that factors such as the carbon chain length, functional groups, and structure (linear and isomeric) of PFASs may affect the binding to HSA. Longer carbon chains could hinder the binding of PFASs to proteins. However, there were different conclusions on the effect of carbon chain length of PFASs on binding to the protein. Previous studies from Li et al. [28] have demonstrated through experiments that various PFASs can be bound to HSA and have found that PFASs have high binding potential with HSA. Alesio et al. [29] analyzed the binding of PFASs to bovine serum albumin (BSA) based upon fluorescence quenching and obtained the binding constants, which are related to the physicochemical properties of PFASs. Recently, MacManus-Spencer et al. [30] compared three experimental methods to detect the binding of PFCAs to serum albumin and found that although fluorescence spectroscopy is an indirect method, it can more comprehensively describe the properties of the interaction. Moreover, Chen et al. [31] also analyzed the binding of PFCAs to HSA using the fluorescence spectroscopy and the interaction forces. Yet, few studies employing the fluorescence spectroscopy are available on the bindings between novel PFASs alternatives and HSA so far.

To better explore the bindings between PFASs and HSAs, the bindings can be modeled using molecular docking techniques. Molecular docking is an effective method to study intermolecular forces, especially for biomolecular complexes, such as the forces between drugs and receptors. Molecular docking can be applied to acquire information about the conformation, binding site, and binding force of the binding between ligand and receptor [32]. The employment of molecular docking techniques not only saves a lot of time, but also provides the binding parameters of the ligand and receptor more quickly and directly. One study used molecular docking to observe the binding of PFOS and HSA and obtained information on the binding energy as well as the binding site, showing the maximum number of ligands that can bind to HSA was 9 for PFOA and 11 for PFOS, and that both of them had the highest binding free energy near the Trp214 binding site [33]. Another researcher utilized molecular docking to explore the mechanism of PFASs transfer through the human placenta. It was shown that the binding affinity of PFASs to HSA increased with growing carbon chain length [34]. So far, most studies have focused on revealing the binding mechanism between legacy PFASs and HSA, yet little information is available about the binding mechanism between novel PFASs alternatives and HSA.

In the present study, three legacy long-chain PFASs, including PFOS, PFOA, and PFHxS, three short-chain PFASs alternatives, including PFPrS, PFBS, and PFBA, together with four novel PFASs alternatives, 9Cl–PF3ONS, 11Cl–PF3OUdS, NaDONA, and HFPO–DA, were adopted as the target legacy and novel PFASs to achieve the following goals: (1) proving experimentally whether 10 PFASs would be bound with HSA and obtaining the information of binding sites and binding constants through the fluorescence spectroscopy; (2) deriving further information of binding sites and binding energy via the molecular docking technique; and (3) based upon all the obtained results, exploring the differences in the bindings of legacy and novel PFASs to HSA.

## 2. Materials and Methods

### 2.1. Chemicals and Reagents

Native PFPrS, PFBS, PFHxS, PFOS, PFBA, PFOA, 9Cl–PF3ONS, 11Cl–PF3OUdS, NaDONA, and HFPO–DA were purchased from Wellington Laboratories (Guelph, ON, Canada). Each PFAS was prepared in anhydrous ethanol as 1 ×${\;10}^{-3}\;$mol/L stock solution. Each stock solution was stored in a refrigerator at 4 °C for use. HSA (99%, Shanghai Macklin Biochemical Technology Company, Shanghai, China) was configured as 1 ×$\;10^{-3\;}$mol/L stock solution with Tris–HCl buffer (pH 7.4, 1 ×${\;10}^{-3}\;$mol/L). Tris–HCl buffer and ethanol were obtained from Fluka (Steinheim, Germany). Milli-Q water was further cleaned using Waters Oasis HLB Plus (225 mg) cartridges (Milford, MA, USA) to remove residual PFASs. All chemicals and reagents were used as received.

### 2.2. Experimental Methods for Three-Dimensional Fluorescence Spectroscopy

Briefly, 5 mL of HSA stock solution was mixed with 1 mL, 3 mL, and 5 mL of PFASs stock solution individually to obtain the concentration ratios of 5:1, 5:3, and 5:5, respectively, at room temperature, and then the mixtures were allowed to stand at the room temperature for 30 min. Then, 4 mL of each was taken, and the three-dimensional fluorescence spectra of the mixtures were measured by a fluorescence spectrophotometer (F–4700, Hitachi, Tokyo, Japan) to determine the optimal concentration for the instrumental analysis. Consequently, the following concentration ratios were selected by comparison. On one side, 5 mL of HSA stock solution was mixed with 1 mL of PFBS, PFHxS, PFOS, PFBA, PFOA, 9Cl–PF3ONS, and 11Cl–PF3OUdS stock solution to obtain a mixture with a concentration ratio of 5:1. On the other side, 5 mL of HSA stock solution was mixed with 3 mL of PFPrS, NaDONA, and HFPO–DA stock solutions to obtain a mixture with a concentration ratio of 5:3. The three-dimensional fluorescence spectra of the HSA stock solution were measured simultaneously. The excitation wavelength (λ_ex_) ranged from 200–340 nm, the emission wavelength (λ_em_) ranged from 270–460 nm, and the excitation and emission wavelength intervals were both 5 nm.

### 2.3. Experimental Methods for Two-Dimensional Fluorescence Spectroscopy

At the room temperature, mixtures were configured with HSA:PFASs concentration ratios of 5:0, 5:1, 5:5, 5:10, 5:15, 5:20, and 5:30, respectively. The mixtures were allowed to stand for 30 min, and then 4 mL of each was taken for measurement using the fluorescence spectrophotometer (F–4700, Hitachi, Japan). The conditions for measuring the fluorescence spectra were as follows: excitation wavelength (λ_ex_) of 275 nm, emission wavelength (λ_em_) range of 285–430 nm, and excitation slit and emission slit of 5 nm.

### 2.4. Molecular Docking Simulation Strategy

The crystal structure of HSA was taken from the Protein Structure Database (Protein Data Bank) with PDB code 4E99 [35]. The structure of PFASs was drawn using Chemdraw 21.0.0. and optimized for the minimum energy to reach the lowest energy state of the chemical structure [36]. Molecular docking simulations of PFASs binding with HSA were performed using Autodock Vina (v 1.1.2) [37]. A grid box of 126–126–126 with a spacing of 0.581 Å was used in order to include all possible binding sites for each PFAS to HSA. The center grid box of x, y, and z was 0.861, 1.278, and −4.167, respectively. Each simulation generated 10 binding modes and the value of exhaustiveness is 10. Molecular docking results were visualized and analyzed using PyMOL 2.5.2. [38].

### 2.5. Data Analysis

The fluorescence quenching data were usually analyzed by Modified Stern–Volmer Equations (1) and (2) [39]

(1) $$\frac{F_{0}}{\left(F_{0}-F\right)}=\frac{1}{K_{SV}^{\prime }f_{a}\left[C\right]}+\frac{1}{f_{a}}$$

(2) $$K_{q}=\frac{K_{SV}^{\prime }}{\tau_{0}}$$

*F_0_* refers to the luminescence intensity of HSA in the absence of PFASs, and *F* refers to the luminescence intensity of HSA in the presence of different amounts of PFASs. $K_{SV}^{\prime }$ is the slope of the modified Stern–Volmer curve, which is the quenching constant of the interacting system. $\tau_{0}$ refers to the average lifetime of the fluorescent substance in the absence of a fluorescent quenching agent. The average fluorescence lifetime of biomolecules is generally 10^−8^ s.

When small molecules bind independently to a set of equivalent sites on a macromolecule, the equilibrium between free and bound molecules is given by the Lineweaver–Burk double logarithmic Equation (3) [40]

(3) $$\mathrm{lg}\left[\frac{\left(F_{0}-F\right)}{F}\right]=\mathrm{lg}K_{A}+n\mathrm{lg}\left[C\right]$$

$K_{A}$ denotes the binding constant of PFASs to HSA, and *n* denotes the number of apparent binding sites between them.

## 3. Results and Discussion

### 3.1. Three-Dimensional Fluorescence Spectroscopy Analysis

**Figure 1:**
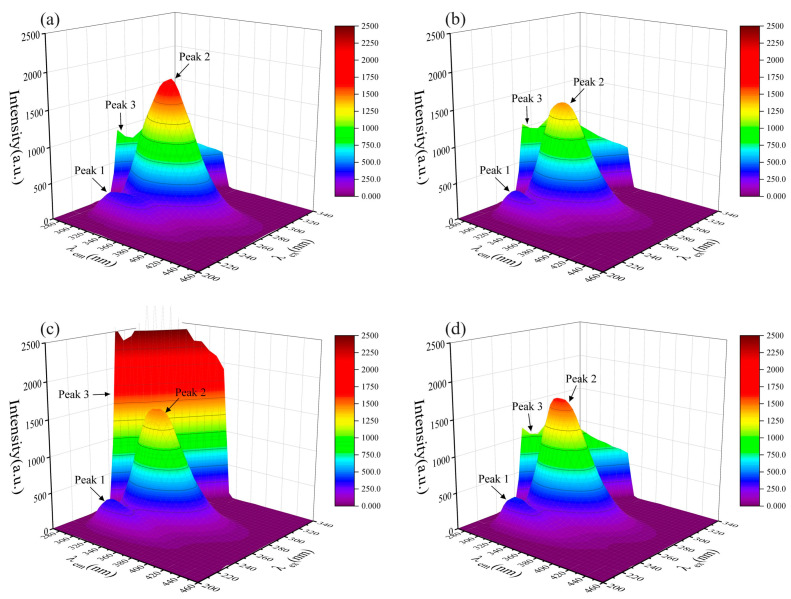
Three-dimensional fluorescence spectrogram for the bindings between HSA and long-chain PFASs: (**a**) HSA, (**b**) HSA–PFOA, (**c**) HSA–PFHxS, (**d**) HSA–PFOS.

Three-dimensional fluorescence spectroscopy can intuitively reflect the protein fluorescence intensity and its conformational change information, which can be used for the analysis of HSA conformation and microenvironment [41]. Two distinct fluorescence peaks (peak 1, λ_em_ = 235 nm, λ_ex_ = 325 nm, I_1_ = 362.3 and peak 2, λ_em_ = 280 nm, λ_ex_ = 335 nm, I_2_ = 1803), together with a first-order Rayleigh scattering peak (peak 3, λ_ex_ = λ_em_ = 290 nm, I_3_ = 1058) can be seen in the three-dimensional fluorescence spectrogram of HSA (Figure 1a). Among them, peak 1 reflects information such as the structure of the protein polypeptide chain, while peak 2 reflects the spectral behavior of the tryptophan and tyrosine residues of HSA [42,43]. The three-dimensional fluorescence spectra of long-chain PFASs before and after binding with HSA are shown in Figure 1b–d.

**Figure 2:**
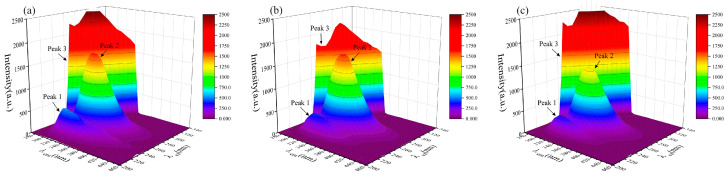
Three-dimensional fluorescence spectrogram for the bindings between HSA and short-chain PFASs alternatives: (**a**) HSA–PFPrS, (**b**) HSA–PFBS, (**c**) HSA–PFBA.

**Figure 3:**
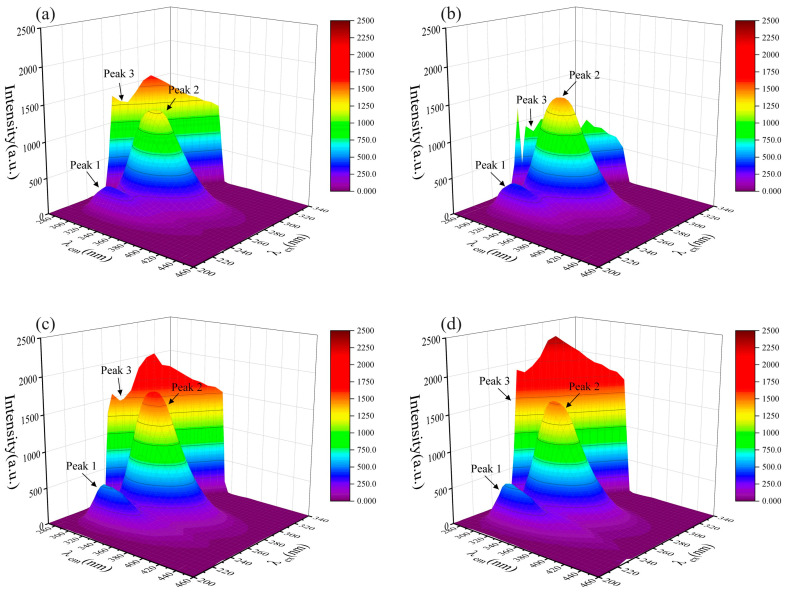
Three–dimensional fluorescence spectrogram for the bindings between HSA and novel PFASs alternatives: (**a**) HSA–9Cl–PF3ONS, (**b**) HSA–11Cl–PF3OUdS, (**c**) HSA–HFPO–DA, (**d**) HSA–NaDONA.

Similarly, the fluorescence spectra of short-chain PFASs bound to HSA and fluorescence spectra of PFASs alternatives bound to HSA were measured as shown in Figure 2 and Figure 3. However, the fluorescence peak intensities of PFPrS, NaDONA, and HFPO–DA did not change significantly. This might be because the binding constants of these three alternatives for long-chain PFASs with HSA were lower, so their concentration ratios needed to be increased to observe the changes. Therefore, the three-dimensional fluorescence spectra of HSA with a concentration ratio of 5:3 of these three PFASs were measured accordingly, and the changes could be clearly observed.

After the addition of each PFAS, the 3D fluorescence spectrogram changed significantly. The intensity of the Rayleigh scattering peak (peak 3) increased due to the growth of solute particle size in the solution. Specifically, the values were I_3, PFOA_ = 1204, I_3, PFOS_ = 1305, I_3, PFHxS_ = 3078, I_3, PFPrS_ = 2622, I_3, PFBS_ = 2238, I_3, PFBA_ = 2832, I_3, 9Cl–PF3ONS_ = 1654, I_3, 11Cl–PF3OUdS_ = 1336, I_3, HFPO–DA_ = 2115, and I_3, NaDONA_ = 2351 individually. There was no significant change in the values of Peak 1, but all the values of Peak 2 decreased to I_2, PFOA_ = 1444, I_2, PFOS_ = 1633, I_2, PFHxS_ = 1499, I_2, PFPrS_ = 1642, I_2, PFBS_ = 1628, I_2, PFBA_ = 1315, I_2, 9Cl–PF3ONS_ = 1286, I_2, 11Cl–PF3OUdS_ = 1454, I_2, HFPO–DA_ = 1674, and I_2, NaDONA_ = 1533 separately, indicating that PFAS interacted with HSA, resulting in a change in the conformation of HSA, which could improve the non-polarity of the microenvironment of the fluorescent molecules in HSA and result in the increasing size of the protein particles [43].

### 3.2. Two-Dimensional Fluorescence Spectral Analysis

The interaction between organic small molecules and proteins can lead to the protein fluorescence quenching phenomenon, which can be divided into two forms, i.e., dynamic quenching and static quenching. Of these, static quenching refers to the phenomenon wherein organic small molecules and proteins combine with each other to form complexes with the help of intermolecular forces, which leads to the phenomenon of weakening of the fluorescence intensity of proteins [44]. Through the calculation of the fluorescence quenching constant, binding constant, and thermodynamic constant, a preliminary judgment can be made on the mutual binding site and the form of force between small molecules and proteins [45].

In order to clarify the mechanism of endogenous fluorescence quenching of HSA by PFASs, the effects of different concentrations of PFASs on the fluorescence spectrum of HSA were investigated. From A to H, the concentration ratios of HSA to PFASs were 5:0, 5:1, 5:5, 5:10, 5:15, 5:20, 5:30, and 0:5, respectively. Under 275 nm light excitation, the intensity of HSA endogenous fluorescence gradually decreased with the increasing concentration of PFASs. Although there was no obvious change in the peak shape, the fluorescence emission peak was blue-shifted from 335 nm to 310 nm. This indicated that the target PFASs interacted with HSA.

**Figure 4:**
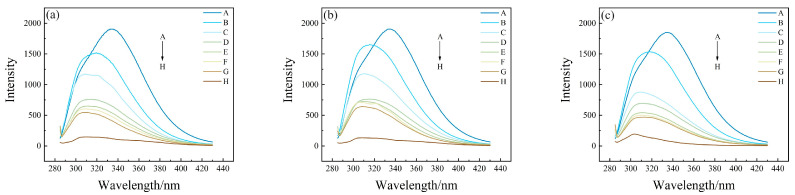
Two-dimensional fluorescence spectrogram for the bindings between HSA and long-chain PFASs: (**a**) HSA–PFOA, (**b**) HSA–PFHxS, (**c**) HSA–PFOS.

**Figure 5:**
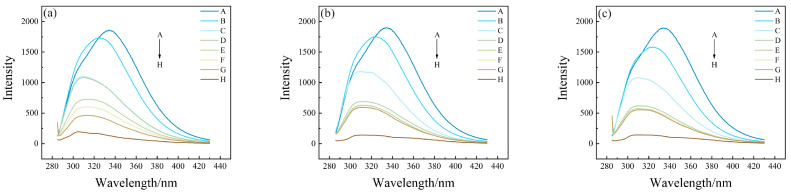
Two-dimensional fluorescence spectrogram for the binding between HSA and short-chain PFASs alternatives: (**a**) HSA–PFPrS, (**b**) HSA–PFBS, (**c**) HSA–PFBA.

**Figure 6:**
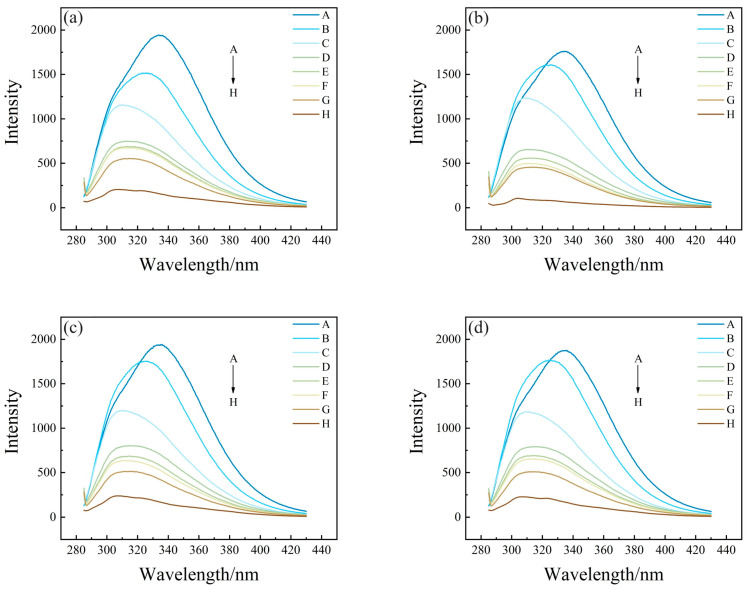
Two-dimensional fluorescence spectrogram for the bindings between HSA and novel PFASs alternatives: (**a**) HSA–9Cl–PF3ONS, (**b**) HSA–11Cl–PF3OUdS, (**c**) HSA–HFPO–DA, (**d**) HSA–NaDONA.

The results of HSA combined with long-chain PFASs, short-chain PFASs, and novel PFASs alternatives are shown in Figure 4, Figure 5, and Figure 6, respectively.

#### 3.2.1. Calculation of Fluorescence Quenching Rate Constants

The fluorescence quenching behavior of HSA was calculated using Equations (1) and (2) [39].

**Figure 7:**
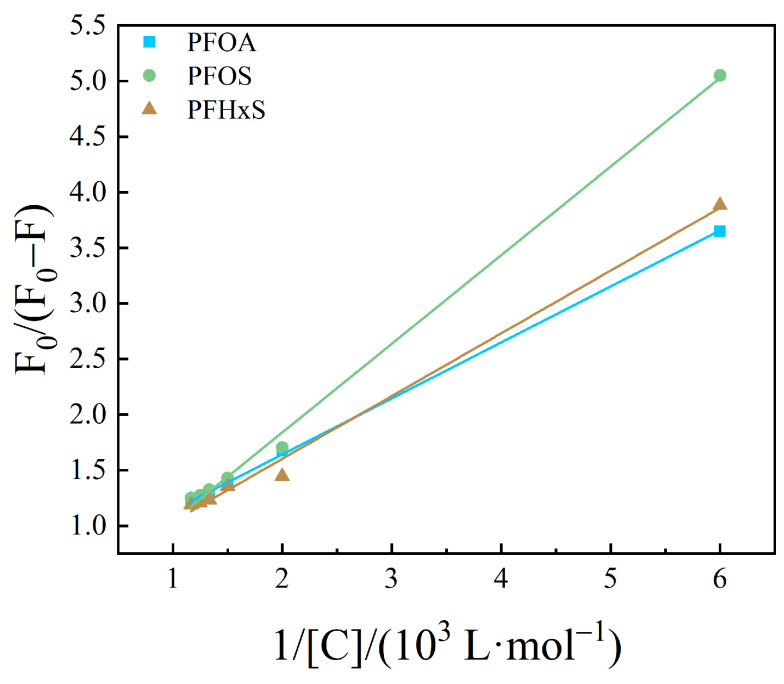
Modified Stern–Volmer equations diagram of HSA combined with long-chain PFASs.

**Figure 8:**
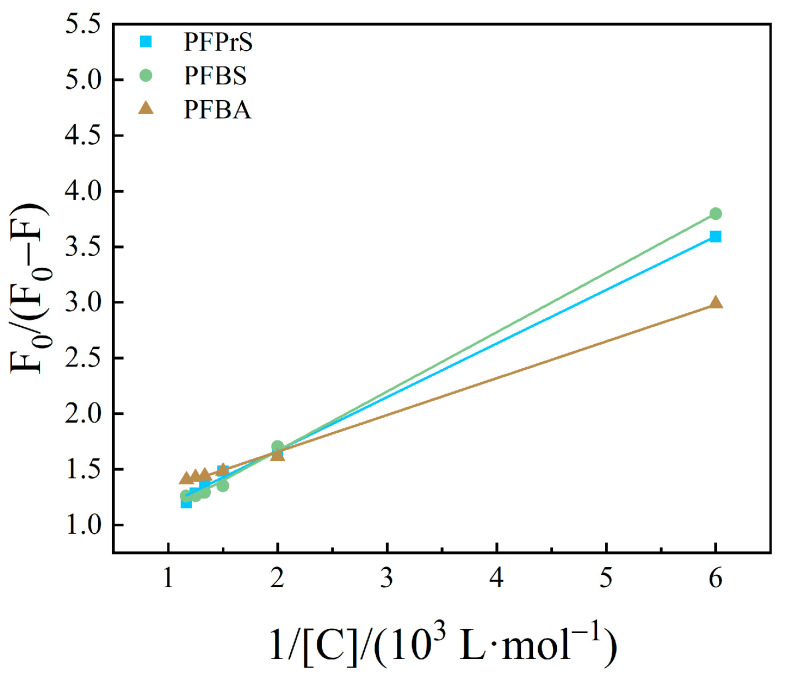
Modified Stern–Volmer equations diagram of HSA combined with short-chain PFASs.

**Figure 9:**
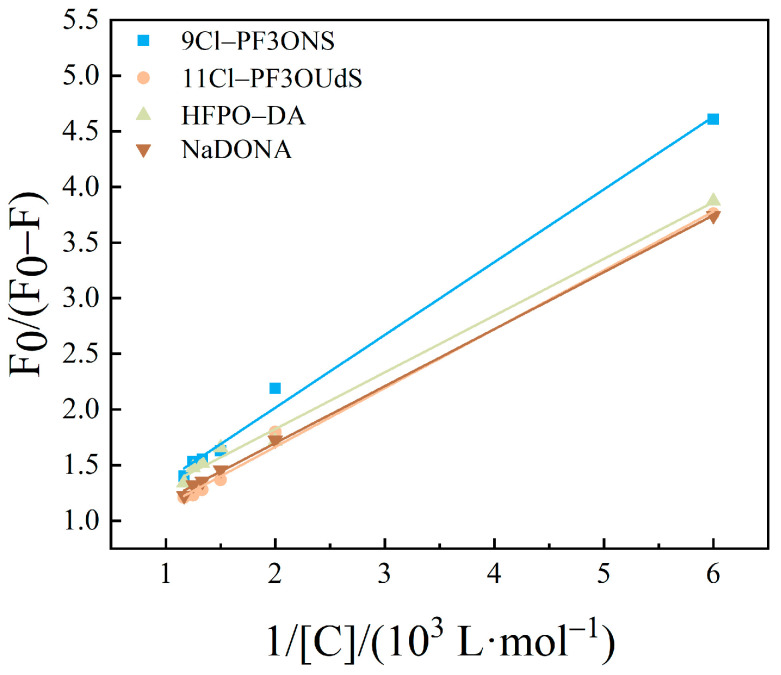
Modified Stern–Volmer equations diagram of HSA combined with novel PFASs alternatives.

The modified Stern–Volmer curves with the inverse concentration of PFASs as the horizontal coordinate and $\frac{F_{0}}{\left(F_{0}-F\right)}$ as the vertical coordinate are shown in Figure 7, Figure 8 and Figure 9. The fitted equations, R^2^, and fluorescence quenching rate constant K_q_ were shown in Table 1, Table 2 and Table 3.

The order of magnitude of *K*_q_ was calculated to be between 10^10^ and 10^11^ in the present study. The maximum scatter collision quenching constant, *K*_q_, of various quenchers with the biopolymer was reported elsewhere to be 2.0 × 10^10^ L/mol/s [46]. Therefore, the fluorescence quenching of PFASs on HSA was not formed by the mutual collision of PFASs on HSA to form a dynamic quenching process, but the formation of a complex might result in a static quenching process [47,48].

#### 3.2.2. Calculation of Binding Sites and Binding Constants

Based upon the results of PFASs binding on HSA as a static quenching process, the binding between the two conforms to the expression formula for the binding of fluorescent substances and quenching agents: Lineweaver–Burk double logarithmic Equation (3) [40]. The binding constants and apparent binding sites were calculated for the system of interaction between PFASs and HSA.

**Figure 10:**
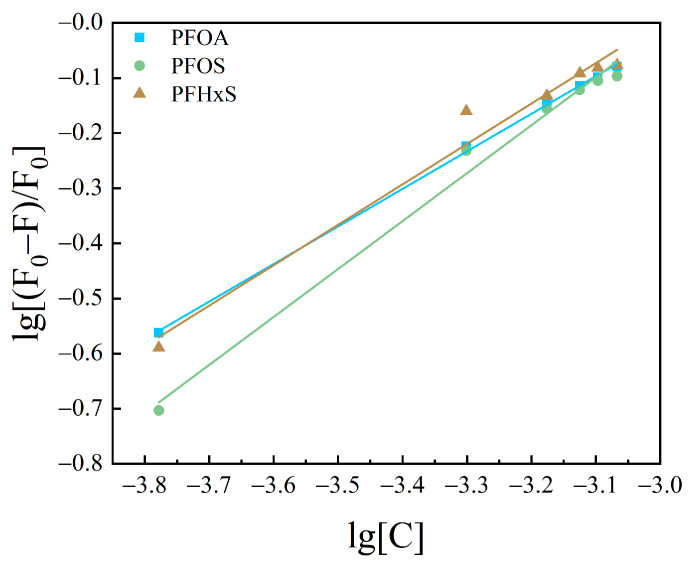
Lineweaver–Burk double logarithmic equation diagram of HSA combined with long-chain PFASs.

**Figure 11:**
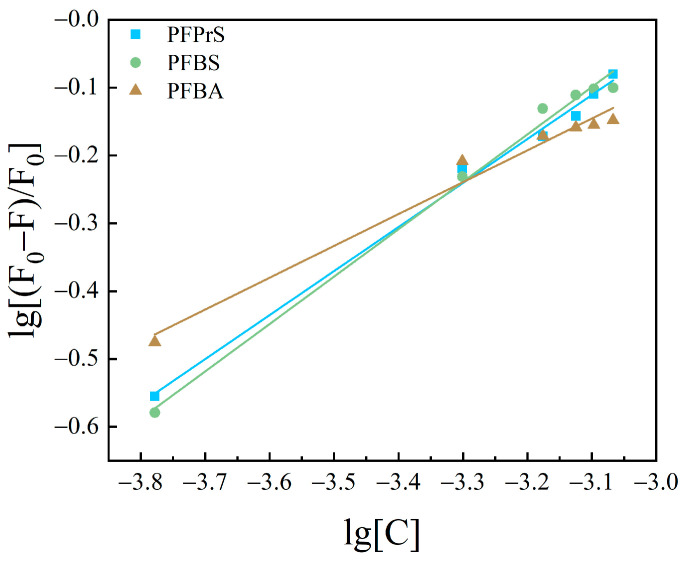
Lineweaver–Burk double logarithmic equation diagram of HSA combined with short-chain PFASs.

**Figure 12:**
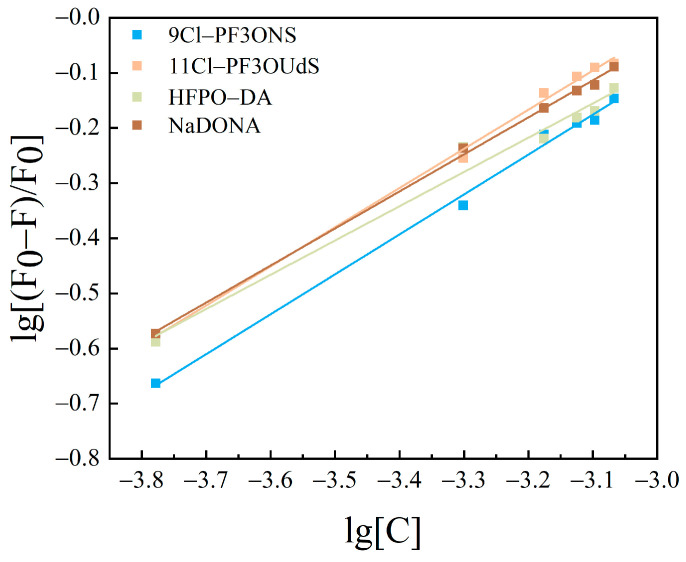
Lineweaver–Burk double logarithmic equation diagram of HSA combined with novel PFASs alternatives.

The curves obtained are shown in Figure 10, Figure 11 and Figure 12. The fitting equations, R^2^, the number of binding sites *n*, and the binding constant *K*_A_ were shown in Table 4, Table 5 and Table 6.

The *n* values of the fitting equations for the binding of HSA to the novel PFASs alternatives were all close to 1, which indicated that one HSA molecule could bind approximately one molecule of the novel PFASs alternatives, and quantitative analyses of the ratio of the two bindings are of great importance for the determination of the forces acting between them [49,50].

According to the results mentioned above, it was demonstrated that the binding constants of PFSAs were greater than those of PFCAs. The binding constants increased with increasing carbon chain length. Among the four novel PFASs alternatives, the binding constants of the sulfonic acid substitutes were higher than those of the carboxylic acid alternatives. 9Cl–PF3ONS and 11Cl–PF3OUdS, as PFOS alternatives, showed that their binding constants with HSA were less than those of PFOS with HSA. Similarly, the binding constants of NaDONA and HFPO-DA, as PFOA substitutes, were significantly smaller than the binding constants of PFOA to HSA. It was suggested that the binding constants of novel PFASs alternatives to HSA were lower than those of legacy PFASs.

### 3.3. Molecular Docking

Some previous studies have used molecular docking simulation techniques to investigate the combination of different PFASs and HSA. Delva-Wiley et al. [51] used molecular docking technology to determine four different binding sites between GenX and HSA. Salvalaglio et al. [33] calculated the number of binding sites between PFOA/PFOS and HSA using the molecular docking techniques. Li et al. [28] calculated the binding affinities of different PFASs and HSA using molecular docking techniques, which can effectively separate PFASs bound to HSA. In this study, molecular docking simulations of HSA with PFASs molecules were performed using Autodock Vina. The conformation with the lowest binding energy for each target PFAS were selected for further analysis.

**Figure 13:**
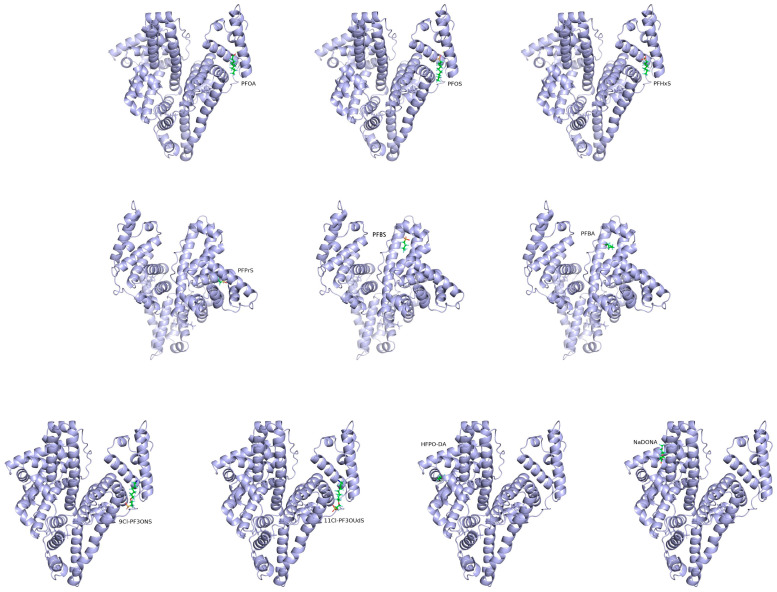
A zoomed-out view of the binding pocket of HSA and long-chain PFASs, short-chain PFASs alternatives, and novel PFASs alternatives.

**Figure 14:**
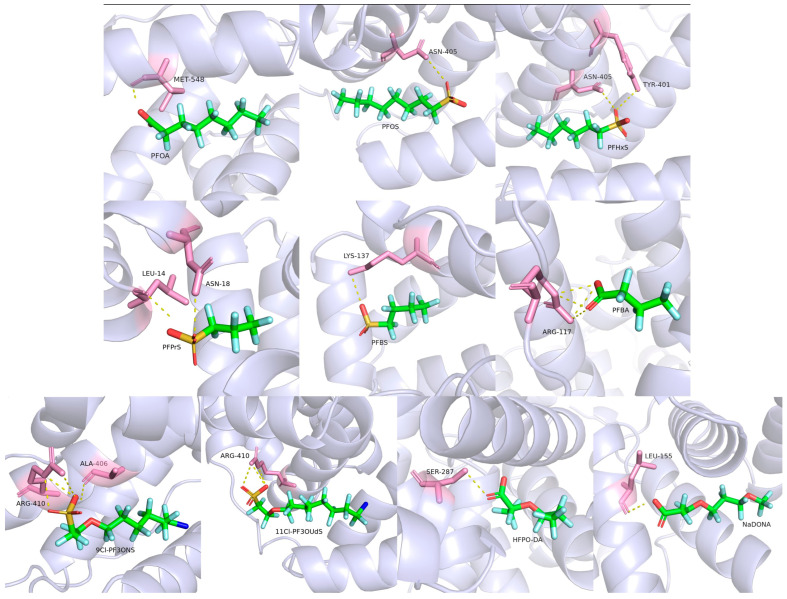
Molecular docking diagram of HSA and long-chain PFASs, short-chain PFASs alternatives, and novel PFASs alternatives.

As shown in Figure 13 and Figure 14, green color represented carbon atoms, cyan color represented fluorine atoms, red color represented oxygen atoms, yellow color represented sulfur atoms, blue represented chlorine atoms, pink color represented amino acid residues, and the yellow dashed line represented hydrogen bonds. Negative figures for the binding energy indicated that the bindings of HSA and PFASs were an exothermic reaction that can occur spontaneously. The larger the absolute value of binding energy, the more energy is released during the synthesis of the complex. Accordingly, the more energy is released, the lower the system energy is retained, and the formed complex is hence more stable [52]. As demonstrated in Table 7, the binding energy between long-chain PFASs and HSA is lower than that of short-chain PFASs alternatives. When the carbon chains are the same, the binding energy of PFSAs is lower than that of PFCAs. As the carbon chain increases, the binding energy decreases. For novel PFASs alternatives, the binding energy of PFOA is lower than its alternatives of NaDONA and HFPO–DA. Similarly, the binding energy of PFOS is lower than its alternatives of 9Cl–PF3ONS and 11Cl–PF3OUdS. Furthermore, the binding energy of short-chain PFASs alternatives is lower than that of novel PFASs alternatives. It is revealed that the complexes between three long-chain PFASs and HSA are more stable than their short-chain and novel alternatives.

Combining the molecular docking results with the experimental results above, it can be summarized that the binding ability of PFSAs to HSA is usually higher than that of PFCAs. Compared to long-chain PFASs, the binding of short-chain PFASs alternatives to HSA is less stable, while the binding ability of novel PFASs alternatives to HSA is lower than that of long-chain PFASs, but higher than that of short-chain PFASs alternatives, which was also described in a previous study [53]. The lower binding affinity of PFASs alternatives shows that they are less likely to be accumulated in the human body. Therefore, it is also suggested that short-chain and novel PFASs alternatives might tend to transport from maternal sera to cord sera through the placenta, due to their lower binding affinity to the placental HSA. Consistently, our recent study based upon the comprehensive analysis of 21 legacy and 49 novel PFASs in 50 paired maternal–infant serum samples around Fuxin fluorochemical facilities determined that legacy PFASs tended to exist in the maternal sera, whereas novel PFASs alternatives tended to exist in the sera of infants [19].

## 4. Conclusions

In summary, the bindings of HSA with legacy and novel PFASs alternatives were initially observed using a three-dimensional fluorescence spectrogram, and the results proved the bindings of HSA and all the target PFASs. The fluorescence quenching mechanism of HSA was then investigated using a two-dimensional fluorescence spectrogram. With the increasing concentration of PFASs, the fluorescence intensity of HSA was gradually decreased and the fluorescence emission peak was blue-shifted from 335 nm to 310 nm, which indicated that the fluorescence quenching of HSA occurred. Moreover, calculations using modified Stern–Volmer equations showed that the quenching constants were greater than 2.0 × 10^10^ L/mol/s, representing that the quenching mechanism of HSA was static quenching. The binding constants as well as the numbers of binding sites were also calculated using the Lineweaver–Burk double logarithmic equation, showing that the binding ratios of all the PFASs to HSA were 1:1. Except for PFPrS, the binding constants of PFSAs and their alternatives were all greater than 10^2^, and the binding constant increased with the growth of the carbon chain. Except for PFOA, the binding constants of PFCAs and their alternatives were all less than 10^2^, and the binding constant declined as the carbon chain decreased. Notably, the binding constants of novel PFASs alternatives were smaller than those of legacy PFASs. Furthermore, the molecular docking technique was used to simulate the bindings between HSA and each target PFAS, revealing that the binding energies between legacy long-chain PFASs and HSA were usually lower than those of short-chain and novel PFASs alternatives, and the binding energies between HSA and PFSAs were usually smaller than those of HSA and PFCAs. It was demonstrated that, compared to legacy long-chain PFASs, short-chain PFASs and novel PFASs alternatives could be less likely to be accumulated in the human body and have higher mobility because of their lower binding affinities to HSA. Consequently, binding to HSA might be considered as an important influencing factor for the bioaccumulation of legacy and novel PFASs. Further research would be warranted to focus on the impact of the bindings of HSA and PFASs on the placental transfer of PFASs and associated health risks of newborns.

## Figures and Tables

**Table 1:** The fitted equations and fluorescence quenching rate constants of long-chain PFASs.

PFAS	Fitted Equation	R^2^	Fluorescence Quenching Rate Constant *K*_q_
PFOA	y = 0.0005035x + 0.635	0.9996	1.26 × 10^11^
PFOS	y = 0.0007969x + 0.2456	0.9977	3.08 × 10^10^
PFHxS	y = 0.0005641x + 0.4741	0.9946	8.40 × 10^10^

**Table 2:** The fitted equations and fluorescence quenching rate constants of short-chain PFASs.

PFAS	Fitted Equation	R^2^	Fluorescence Quenching Rate Constant *K*_q_
PFPrS	y = 0.0004813x + 0.7054	0.9977	1.47 × 10^11^
PFBS	y = 0.0005327x + 0.6013	0.9989	1.13 × 10^11^
PFBA	y = 0.0003302x + 0.9979	0.9985	3.02 × 10^11^

**Table 3:** The fitted equations and fluorescence quenching rate constants of novel PFASs alternatives.

PFAS	Fitted Equation	R^2^	Fluorescence Quenching Rate Constant *K*_q_
9Cl-PF3ONS	y = 0.0006538x + 0.7081	0.9948	1.08 × 10^11^
11Cl-PF3OUdS	y = 0.0005285x + 0.6068	0.9956	1.15 × 10^11^
HFPO-DA	y = 0.0005095x + 0.8051	0.9945	1.58 × 10^11^
NaDONA	y = 0.0005118x + 0.6743	0.9994	1.32 × 10^11^

**Table 4:** The fitted equations, binding constants, and the numbers of binding sites of long-chain PFASs.

PFAS	Fitted Equation	R^2^	Binding Constant *K*_A_	The Number of Binding Sites *n*
PFOA	y = 0.6811x + 2.0143	0.9991	103.3475	0.6811
PFOS	y = 0.8694x + 2.5962	0.9896	394.639	0.8694
PFHxS	y = 0.7328x + 2.1985	0.9754	157.9429	0.7328

**Table 5:** The fitted equations, binding constants, and the numbers of binding sites of short-chain PFASs.

PFAS	Fitted Equation	R^2^	Binding Constant *K*_A_	The Number of Binding Sites *n*
PFPrS	y = 0.6487x + 1.9001	0.9939	79.4511	0.6487
PFBS	y = 0.6986x + 2.0663	0.9931	116.4930	0.6986
PFBA	y = 0.4692x + 1.3089	0.9792	20.3657	0.4692

**Table 6:** The fitted equations, binding constants, and the numbers of binding sites of novel PFASs alternatives.

PFAS	Fitted Equation	R^2^	Binding Constant *K*_A_	The Number of Binding Sites *n*
9Cl-PF3ONS	y = 0.724x + 2.0686	0.9952	117.112	0.724
11Cl-PF3OUdS	y = 0.7102x + 2.1059	0.9966	127.6145	0.7102
HFPO-DA	y = 0.6214x + 1.7708	0.9794	58.9929	0.6214
NaDONA	y = 0.6718x + 1.9689	0.9982	93.0894	0.6718

**Table 7:** Simulated binding energies and binding residues of HSA to three types of PFASs via molecular docking.

Target PFAS	Binding Energy (kcal/mol)	Hydrogen Bonds	Binding Residues
Long-chain PFASs			
PFOA	−7.6	1	MET548
PFOS	−8.3	1	ASN405
PFHxS	−7.5	2	ASN405, TYR401
Short-chain PFASs alternatives			
PFBA	−6.2	4	ARG117
PFBS	−6.4	1	LYS137
PFPrS	−6.2	2	LEU14, ASN18
Novel PFASs alternatives			
NaDONA	−7.3	1	LEU155
HFPO-DA	−7.0	1	SER287
9Cl-PF3ONS	−7.8	4	ARG410, ALA406
11Cl-PF3OUdS	−7.9	3	ARG410

## Data Availability

Data are contained within the article.
